# Cardiometabolic factors related to post-COVID-19 conditions: a scoping review

**DOI:** 10.15649/cuidarte.4290

**Published:** 2025-07-24

**Authors:** João Cruz Neto, Carlos Vinícius Fiuza Olivindo, José Arthur Guimarães dos Santos, María Alejandra Araujo da Silva, Romulo de Oliveira Sales Junior

**Affiliations:** 1 Universidade Federal do Ceará – UFC. Fortaleza, Ceará, Brazil. E-mail: enfjcncruz@gmail.com UFC Ceará Brazil enfjcncruz@gmail.com; 2 Universidade Federal do Ceará – UFC. Fortaleza, Ceará, Brazil. E-mail: viniciusolivindo@gmail.com UFC Ceará Brazil viniciusolivindo@gmail.com; 3 Universidade Federal da Paraíba - UFPB. João Pessoal, Paraíba, Brazil. E-mail: arthurguimaraes60@gmail.com UFPB Paraíba Brazil arthurguimaraes60@gmail.com; 4 Universidade Federal do Ceará – UFC. Fortaleza, Ceará, Brazil. E-mail: kadsonp64@gmail.com UFC Ceará Brazil kadsonp64@gmail.com; 5 Universidade Estadual Paulista - UNESP. São Paulo, Brazil. E-mail: romulojr_99@hotmail.com UNESP São Paulo Brazil romulojr_99@hotmail.com

**Keywords:** Adult, COVID-19, Syndrome, Cardiometabolic Risk Factors, Adulto, COVID-19, Síndrome, Factores de Riesgo Cardiometabólico, Adulto, COVID-19, Síndrome, Fatores de Risco Cardiometabólico

## Abstract

**Introduction:**

Post-COVID syndrome is a pathology that involves multiple sequelae. It is important to identify cardiometabolic risk factors as a way of preventing complications.

**Objective:**

To map the scientific evidence related to cardiometabolic factors in long post-COVID-19 conditions.

**Materials and Methods:**

Scoping review with the guiding question: What scientific evidence relates cardiometabolic factors to patients with long post-Covid-19 syndrome? The sources of information used were six databases via the CAPES journal portal. For the gray literature, we used the CAPES catalog of theses and dissertations, the Brazilian Digital Library of Theses and Dissertations, the Who Library Database and the medRxiv and OpenGrey repositories. The following descriptors were used: Adult, heart disease risk factors, Syndrome, SARS-CoV-2 and Covid 19 crossed using the Boolean operators AND and OR.

**Results:**

14 studies were included. The cardiometabolic factors found were: abnormal levels of triglycerides, glycated hemoglobin, ferritin, inflammatory processes, decreased platelets, phospholipids and endothelial cells, oxidative stress, higher concentrations of monosaccharides and reduced polysaccharides, increased LDL, ALT, AST and bilirubin, with reduced GFR.

**Discussion:**

Patients with long-term COVID report persistent and debilitating symptoms that affect recovery, quality of life, economic and social activities. In addition to increased resting heart rate, tachycardia, palpitations, hypotension, syncope, orthostatic tachycardia, angina and heart attack.

**Conclusion:**

Cardiometabolic factors expose the vulnerability of individuals affected by long Covid-19, so strategies are needed to reduce the systemic inflammatory impact of the disease and its clinical consequences.

## Resumen

**Introducción:**

El síndrome post-COVID es una patología que conlleva múltiples secuelas. Es importante identificar los factores de riesgo cardiometabólicos para prevenir las complicaciones de la condición clínica.

**Objetivo:**

Mapear la evidencia científica relacionada con los factores cardiometabólicos en afecciones de larga duración post-COVID-19.

**Materiales y métodos:**

Revisión exploratoria con la pregunta guía: ¿Qué evidencia científica relaciona los factores cardiometabólicos con los pacientes con síndrome post-COVID-19 de larga duración? Las fuentes de información utilizadas fueron seis bases de datos del portal de revistas CAPES. Para la literatura gris, se utilizaron el catálogo de tesis y disertaciones de CAPES, la Biblioteca Digital Brasileña de Tesis y Disertaciones, la base de datos de la Biblioteca de la OMS y los repositorios medRxiv y OpenGrey. Los descriptores utilizados fueron: adulto, factores de riesgo de cardiopatía, Síndrome, SARS-CoV-2 y Covid-19, cruzados mediante los operadores booleanos AND y OR.

**Resultados:**

Se incluyeron catorce estudios. Los factores cardiometabólicos encontrados fueron: niveles anormales de triglicéridos, hemoglobina glucosilada, ferritina, procesos inflamatorios, disminución de plaquetas, fosfolípidos y células endoteliales, estrés oxidativo, mayores concentraciones de monosacáridos y reducción de polisacáridos, aumento de LDL, ALT, AST y bilirrubina, con reducción de GFR.

**Discusión:**

Los pacientes con COVID de larga duración reportan síntomas persistentes y debilitantes que afectan la recuperación, calidad de vida, actividades económicas y sociales. Además de aumento de la frecuencia cardíaca en reposo, taquicardia, palpitaciones, hipotensión, síncope, taquicardia ortostática, angina e infarto.

**Conclusiones:**

Los factores cardiometabólicos exponen la vulnerabilidad de los individuos afectados por COVID-19 de larga duración, por lo que se necesitan estrategias para reducir el impacto inflamatorio sistémico de la enfermedad y sus consecuencias clínicas.

## Resumo

**Introdução:**

A síndrome pós-COVID é uma patologia que envolve múltiplas sequelas. Torna-se relevante a identificação dos fatores de risco cardiometabólicos como forma de prevenir complicações do quadro clínico.

**Objetivo:**

Mapear as evidências científicas relacionadas aos fatores cardiometabólicos em condições pós-Covid-19 longa.

**Materiais e métodos:**

Revisão de escopo com a questão norteadora: Quais as evidências científicas que relacionam os fatores cardiometabólicos a pacientes com a síndrome pós-Covid-19 longa? As fontes de informação utilizadas foram seis bases de dados via portal de periódicos da CAPES. Para a literatura cinzenta empregou-se catálogo de teses e dissertações da CAPES, Biblioteca Digital Brasileira de Teses e Dissertações, Who Library Database e os repositórios medRxiv e OpenGrey. Foram utilizados os descritores: adult, heart disease risk factors, Syndrome, SARS-CoV-2 e Covid-19 cruzados por meio dos operadores booleanos AND e OR.

**Resultados:**

Foram incluídos 14 estudos. Os fatores cardiometabólicos encontrados foram: níveis anormais de triglicerídeos, hemoglobina glicada, ferritina, processos inflamatórios, diminuição de plaquetas, fosfolipídios e células endoteliais, estresse oxidativo, maiores concentrações de monossacarídeos e redução de polissacarídeos, aumento de LDL, ALT, AST e bilirrubina, com redução de TFG.

**Discussão:**

Os pacientes com COVID de longa duração relatam sintomas persistentes e debilitantes que afetam a recuperação, qualidade de vida, atividades econômicas e sociais. Além do aumento da frequência cardíaca em repouso, taquicardia, palpitações, hipotensão, síncope, taquicardia ortostática, angina e ataque cardíaco.

**Conclusões:**

Os fatores cardiometabólicos expõem a vulnerabilidade dos indivíduos acometidos pela Covid-19 longa, assim, são necessárias estratégias que reduzam o impacto inflamatório sistêmico da doença e suas consequências clínicas.

## Introduction

Risk factors constitute a group of clinical implications that can lead to illness. In this sense, cardiometabolic factors are related to a worse clinical prognosis in circulatory, respiratory or metabolic origin, with clinical implications related to hypercytokinemia, inflammation, severe acute respiratory syndrome, abdominal adiposity, cardiovascular diseases, hypercoagulability and hydroelectrolytic imbalances^1^,^2^.

Lower-than-expected immune responses in patients with metabolic syndrome occur because of the pro-inflammatory environment, generating hyperinflammation, microvascular dysfunction, and cardiovascular events. As a result, patients with cardiometabolic comorbidities have a higher risk of unfavorable outcomes^3^.

Although COVID-19 infection is directly related to respiratory dysfunction, other complications have been elucidated, including cardiovascular system dysfunction^4^. Patients infected with SARS-CoV-2 developed cardiac complications, some of which were the worsening of pre-existing pathologies and others developed after contact with the virus. In addition, it was noticeable that older men, smokers and those with comorbidities such as Systemic Arterial Hypertension, Diabetes Mellitus and other heart diseases are part of a group more susceptible to adverse outcomes^5^.

“Post-COVID conditions” can be defined as clinical conditions developed or developing after COVID-19 infection that imply a worsening of the health status and that cannot be attributed to other causes^6^. Furthermore, the use of codes from the International Statistical Classification of Diseases and Related Health Problems of the 10th Revision (ICD-10) is recommended as a way of recording post-COVID conditions, with code U09.9 being indicated with the description of an unspecified post-COVID-19 health condition^7^.

Different phases of COVID-19 are observed: acute COVID-19 (sequelae for up to 4 weeks), acute post-COVID symptoms: (4 to 12 weeks) and long post-COVID-19 syndrome (occur during or after an infection consistent with COVID-19, continuous for more than 12 to 24 weeks and are not explained by an alternative diagnosis), persistent Post-COVID symptoms (greater than 24 weeks)^8^,^9^.

Post-COVID syndrome is a pathology that involves persistent physical, mental and cognitive sequelae after becoming ill between three and six months after the onset of the first symptom. Patients who were no longer positive for SARS-CoV-2 and were discharged from the hospital, as well as outpatients, can also develop long COVID^10^.

Therefore, it is important to identify cardiometabolic risk factors to prevent complications of the clinical condition. To this end, health professionals must pay attention to the specific signs of cardiac, vascular and metabolic diseases, although this point is still a deficit in health care^11^.

This review becomes necessary, given the relevance of the topic, scope and need to identify cardiometabolic factors to elucidate health care practices at different levels of care, in addition to promoting the prevention of injuries and promotion of care.

After a search in Medical Literature Analysis and Retrieval System Online (MEDLINE), Open Science Framework, JBI Evidence Synthesis, Cochrane Database of Systematic Reviews No ongoing or conducted reviews were found on factors and conditions associated with post-long Covid-19. The objective of this review is to map the scientific evidence related to cardiometabolic factors in post-long Covid-19 conditions.

## Materials and Methods

**Type of study**

This is a scoping review that was developed in accordance with the evidence synthesis manual published by JBI^12^ and guided by the methodological guidelines proposed by the PRISMA extension (Preferred Reporting Items for Systematic reviews and Meta- Analyses) for scoping reviews, PRISMA-ScR^13^. The study was registered on the Open Science Framework platform. The study data are available for free access on the Mendeley Data platform^14^.

**Study stages**

The review was developed through nine sequential steps, namely: (1) Determining objective and question; (2) Formulating inclusion criteria; (3) Directing research planning, selection, data extraction and presentation of evidence; (4) Data mapping; (5) Evidence selection; (6) Evidence extraction; (7) Evidence analysis; (8) Presentation of results; and (9) Applying the relationship between objective, conclusion and implications of the findings^12^.

**Research question**

The research question was developed using the PCC strategy, with “P” being the population - adult patients with long post-COVID-19 syndrome”, “C” the concept - cardiometabolic factors after long post-COVID-19, and the second “C” the context - Various health scenarios. Thus, the following guiding research question was established: What scientific evidence relates cardiometabolic factors to patients with long post-COVID-19 syndrome?

**Eligibility criteria**

Studies with patients over 18 years of age with long post-COVID-19 syndrome and some cardiometabolic disease were included. Studies in which there was a worsening of the health condition unrelated to COVID-19 or interaction with cardiometabolic disease in their population were excluded.

The study included people with cardiometabolic diseases or who developed them after infection with Covid-19. Cardiometabolic factors such as oxidative stress, endothelial dysfunction, insulin resistance, atherosclerosis, increased body fat and changes in the microbiome are understood to be influenced or not by Covid-19.

Studies in different health settings (outpatient, home, hospital) were included without limitations by geographic location or social, ethnic or gender factors.

Studies with different primary methodological designs and without a time frame were included. Editorial studies, narrative reviews, abstracts in annals, research projects and protocols were excluded. Studies that answered the guiding question of this review were read in full and the references were analyzed in search of additional studies for potential inclusion. Studies that were not related to the objectives of the review were excluded, based on reading the title and abstract, unassociated themes, availability in fully broad research, analysis of repeated studies, in addition to reading and evaluating the findings for irrelevant content.

**Sources of information**

Sources of information: Latin American and Caribbean Literature on Health Sciences (LILACS); Medical Literature Analysis and Retrieval System Online (MEDLINE) via PubMed; Web of Science (WoS), Embase via Elsevier; EBSCO (FSTA - Food Science and Technology) and SCOPUS via CAPES journal portal. For gray literature, the CAPES theses and dissertations catalog, Brazilian Digital Library of Theses and Dissertations (BDTD), Who Library Database and the medRxiv and OpenGrey repositories were used. The search for studies was carried out between March and June 2023 in a paired manner.

**Search Strategies**

The search strategy for articles was carried out based on the PCC elements with terms used in the descriptors in Health Sciences (DeCS) or Medical Subject Headings (MeSH), together with the Boolean operators AND and OR. The best search strategy was selected according to the tests carried out on the PubMed portal, considering the strategy that resulted in a greater number of studies related to the theme proposed by the review.

**Table 1:** Research strategy, 2023

Bibliographic Sources	Research Strategy
MEDLINE 364 [March 21 at 9:35 pm]	((adult[MeSH Terms]) AND (((((((((((((((((((((((((((((((((((((((((((((((((((((((((((((cardiometabolic risk factors[MeSH Terms]) AND (metabolic syndrome[MeSH Terms]))) OR (cardiometabolic syndrome[Title/Abstract])) OR (cardiovascular syndrome metabolic[Title/Abstract])) OR (metabolic cardiovascular syndrome[Title/Abstract])) OR (metabolic x syndrome[Title/Abstract])) OR (syndrome cardiometabolic[Title/Abstract])) OR (syndrome metabolic cardiovascular[Title/Abstract])) OR (syndrom)) OR (syndromal)) OR (syndromally)) AND (Syndrome[MeSH Terms]))) OR (syndromes)) OR (Cardiometabolic[Title/Abstract])) AND (risk factors[MeSH Terms])) OR (correlates health[Title/Abstract])) OR (risk factor[Title/Abstract])) OR (risk factor score[Title/Abstract])) AND (heart disease risk factors[MeSH Terms])) AND (COVID-19[MeSH Terms])) OR (2019 ncov disease[Title/Abstract])) OR (2019 ncov infection[Title/Abstract])) OR (coronavirus disease 2019[Title/Abstract])) OR (covid 19 pandemic[Title/Abstract])) OR (Infection[Title/Abstract])) AND (SARS-CoV-2[MeSH Terms])) OR (associated conditions[Title/Abstract])) OR (associated disease[Title/Abstract])) OR (coexistent conditions[Title/Abstract])) OR (coexistent disease[Title/Abstract])) OR (concomitant conditions[Title/Abstract])) OR (concomitant disease[Title/Abstract])) OR (sequelae[Title/Abstract])) OR (sequel*[Title/Abstract])) OR (covid 19 sequalae[Title/Abstract])) OR (core outcome set[Title/Abstract])) OR (long-COVID[Title/Abstract])) OR (Post-COVID-19[Title/Abstract])) OR (post acute sequelae of sars cov 2 infection[Title/Abstract])) OR (Post-COVID-19[Title/Abstract])) OR (chronic covid syndrome[Title/Abstract])) OR (chronic covid 19[Title/Abstract])) OR (covid long hauler[Title/Abstract])) OR (long haul covid[Title/Abstract])) OR (long hauler covid[Title/Abstract])) OR (post covid 19 fatigue[Title/Abstract])) OR (post covid 19 neurological syndrome[Title/Abstract])) OR (post covid 19 syndrome[Title/Abstract])) OR (post covid fatigue[Title/Abstract])) OR (post covid syndrome[Title/Abstract])) OR (post acute covid syndrome[Title/Abstract])) OR (post acute covid 19[Title/Abstract])) OR (post acute covid 19 syndrome[Title/Abstract])) OR (post acute covid 19 syndrome[Title/Abstract])) OR (long-COVID[Title/Abstract])) OR (long haul covid[Title/Abstract])) OR (persistent covid 19[Title/Abstract])) ) OR (post acute covid19 syndrome[Title/Abstract]))) AND (((((((((((((((((((((((((((((((((ambulatory care[MeSH Terms]) OR (care ambulatory[Title/Abstract])) OR (care outpatient[Title/Abstract])) OR (care*[Title/Abstract])) OR (Urgent[Title/Abstract])) OR (clinic visit*[Title/Abstract])) OR (health service outpatient[Title/Abstract])) OR (outpatient care[Title/Abstract])) OR (outpatient health service[Title/Abstract])) OR (outpatient service*[Title/Abstract])) OR (Service*[Title/Abstract])) OR (servicing[Title/Abstract])) OR (outpatient health[Title/Abstract])) OR (services outpatient[Title/Abstract])) OR (services outpatient health[Title/Abstract])) OR (urgent care*[Title/Abstract])) AND (home nursing[MeSH Terms])) OR (Care)) OR (Care)) OR (nonprofessional home[Title/Abstract])) AND (home environment[MeSH Terms])) OR (Home)) OR (environment)) OR (Home)) OR (care non professional[Title/Abstract])) AND (home environment[MeSH Terms])) OR (Home)) OR (care non professional[Title/Abstract])) AND (home environment[MeSH Terms])) OR (home environment)) OR (care nonprofessional[Title/Abstract])) OR (nonprofessional home care[Title/Abstract])) OR (nursing home[Title/Abstract]))
SCOPUS 144	( ALL ( adult ) AND ALL ( cardiometabolic AND risk AND factors OR metabolic AND syndrome OR risk AND factor* OR heart AND disease AND risk AND factors OR long-covid OR post-covid-19 OR post AND acute AND sequelae OR post AND covid 19 fatigue AND post OR covid 19 syndrome OR sequel* OR chronic AND covid 19 OR post AND covid 19 neurological OR post AND covid AND fatigue OR post AND acute AND covid AND syndrome OR post AND acute AND covid 19 OR persistent AND covid 19 ) )
LILACS 0	adult [Palavras] and cardiometabolic risk factors OR metabolic syndrome OR risk factor* OR heart disease risk factors OR long-COVID OR Post-COVID-19 OR post acute sequelae OR post covid 19 fatigue post OR covid 19 syndrome OR sequel* OR chronic covid 19 OR post covid 19 neurological OR post covid fatigue OR post acute covid syndrome OR post acute covid 19 OR persistent covid 19 [Words]
EMBASE 45	('adult'/exp OR adult) AND ((((((((((((cardiometabolic AND risk AND factors OR metabolic) AND syndrome OR risk) AND factor* OR heart) AND disease AND risk AND factors OR 'long covid' OR 'post covid 19' OR post) AND acute AND sequelae OR post) AND covid AND 19 AND fatigue AND post OR covid) AND 19 AND syndrome OR sequel* OR chronic) AND covid AND 19 OR post) AND covid AND 19 AND neurological OR post) AND covid AND fatigue OR post) AND acute AND covid AND syndrome OR post) AND acute AND covid AND 19 OR persistent) AND covid AND 19 AND ((((ambulatory AND care OR outpatient) AND health AND service OR outpatient) AND service OR urgent) AND care OR nonprofessional) AND home AND care
EBSCO (FSTA - Food Science and Technology) 312	adult AND ( cardiometabolic risk factors OR metabolic syndrome OR risk factor* OR heart disease risk factors OR long-COVID OR Post-COVID-19 OR post acute sequelae OR post covid 19 fatigue post OR covid 19 syndrome OR sequel* OR chronic covid 19 OR post covid 19 neurological OR post covid fatigue OR post acute covid syndrome OR post acute covid 19 OR persistent covid 19 ) AND ( ambulatory care OR outpatient health service OR outpatient service OR urgent care OR nonprofessional home care )
Web Of Science 46	adult (Topic) AND cardiometabolic risk factors OR metabolic syndrome OR risk factor* OR heart disease risk factors OR long-COVID OR Post-COVID-19 OR post acute sequelae OR post covid 19 fatigue post OR covid 19 syndrome OR sequel* OR chronic covid 19 OR post covid 19 neurological OR post covid fatigue OR post acute covid syndrome OR post acute covid 19 OR persistent covid 19 (Topic) AND ambulatory care OR outpatient health service OR outpatient service OR urgent care OR nonprofessional home care (Topic)
CTDC 3613 (2019-2023)	**Area of knowledge: **health sciences. **Area of knowledge: **nursing, medicine, clinical medicine, anatomy, clinical pathology; gynecology and obstetrics. adult and cardiometabolic risk factors OR long-COVID OR Post-COVID-19 OR post acute sequelae OR post covid 19 fatigue post OR covid 19 syndrome OR sequel* OR chronic covid 19 OR post covid 19 neurological OR post covid fatigue OR post acute covid syndrome OR post acute covid 19 OR persistent covid 19^.
BTDC	Adults AND Post-Covid-19 AND cardiometabolic
MedRxiv 27	Adult and cardiometabolic risk factors AND long-COVID OR Post-COVID-19 OR post acute sequelae OR sequel* OR post acute covid
OpenGrey 7	Adult and cardiometabolic risk factors AND long-COVID OR Post-COVID-19

**Study Selection**

For the selection of evidence, a model adapted according to JBI^12^ was used. The results obtained with the search were exported to the Rayyan reference manager developed by Qatar Computing. Research Institute (QCRI)^15^, the selection of studies occurred independently by two researchers; it is worth noting that a third researcher decided conflicts in the absence of consensus. Duplicates were considered only once with the help of the Mendeley SoftwareⓇ.

**Data Extraction**

Extraction was carried out by screening the full text of the included articles: data from the articles were extracted by reading the full text and organized in a spreadsheet that was constructed based on bibliographic information, country and year of publication, type of method adopted, as well as the results related to the research question elaborated based on the PCC proposed for this review.

**Presentation of Results**

**Figure 1:**
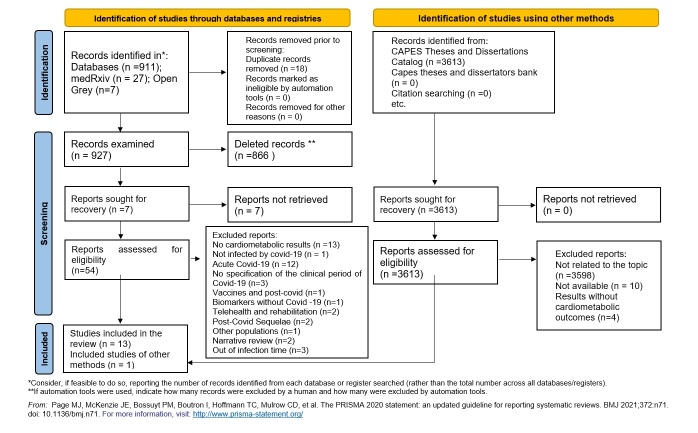
Search and selection of studies included in the review

The results were shown in PRISMA^16^ and mapped in the form of tables/diagrams, Figure 1. These results were accompanied by the preparation of a narrative synthesis of the data, which was constructed according to thematic categories that appeared when reading the selected texts.

## Results

The flow regarding the inclusion process, focusing on the pre-established eligibility criteria of the studies selected for this review, are shown in Figure 1. Fourteen studies were included, from nine different countries, namely: England (n=3, 21.42%), Brazil and the United States (n=2, 14.28%, respectively), Russia, Spain, Germany, Canada, Austria, India and Poland (n=1, respectively).

The study designs were cohort (n=6, 42.85%), observational, systematic reviews and descriptive (n=2, 14.28%, respectively), documentary and clinical trial (n=1, 7.14%, respectively). The sample participants between the studies ranged from 32 to 1357518 people, with an average of 111 for descriptive studies, 567 in observational studies and 3331 in cohort studies. The follow-up time of the studies ranged from 12 to 52 months. The manifestation of symptoms among cardiometabolic factors ranged from 13 weeks in clinical and documentary studies, 46 weeks in observational studies, 65 weeks in descriptive studies and 120 weeks in cohort studies.

**Metabolic implications**

Studies have shown metabolic implications in post-COVID-19 syndrome associated with obesity with increased body fat, predisposed to infectious diseases, progression of pathological severity and cardiometabolic diseases^17^-^21^, leading to symptoms such as weakness, exercise intolerance, palpitations, memory and concentration disorders, chest pain and arthralgia^18^. Abnormal levels of triglycerides, glycated hemoglobin^17^,^18^ and ferritin that can increase the glycemic rate and induce insulin resistance^17^ being responsible for severe cases of prolonged COVID-19^17^,^18^. The risk of developing diabetes mellitus remains high^22^,^23^ after 12 weeks of discharge^24^.

**Circulatory implications**

There is also an increase in inflammatory processes^18^, leading to endothelial injury^23^,^25^–^27^, including hypertension^23^ myocardial injury, myocarditis, arrhythmias, acute coronary syndrome, venous thromboembolism, and heart failure among 60% to 78% of patients in the months following COVID-19 infection^27^–^28^, myocardial injury, abnormal ventricular function, edema, coronary heart disease, ischemic heart disease, valvular anomaly, pericardial effusion, atrial fibrillation, and diastolic dysfunction^27^.

**Respiratory implications**

**Figure 2:**
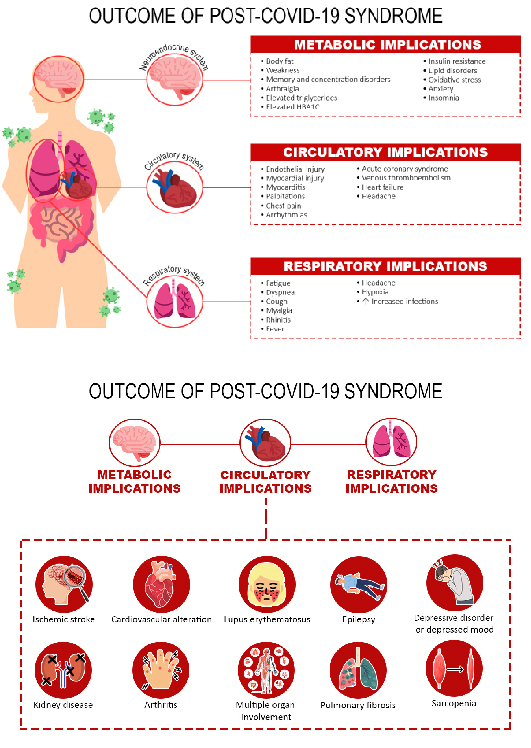
Outcomes and implications of post-covid-19 syndromes

*Note: Adapted from Ely et al.^31^*

Residual cardiovascular system disorders were noted after 6 to 12 weeks, such as fatigue, dyspnea, headache, cough, dysgeusia, fever, and dyspnea^21^,^28^,^29^ myalgia, rhinitis, and fever due to respiratory infections^21^,^23^ pulmonary thromboembolism, heart failure, stroke, coronary heart disease^24^,^28^-^29^, pulmonary embolism, atrial arrhythmia, and venous thrombosis^22^,^24^, and myocardial infarction^23^-^24^. Oxidative stress leads to high levels of fatty acids, MUFA, and low PUFA in the blood, which are associated with cardiovascular risks, metabolic diseases, and infections^30^. The relationship between the implications, factors, and outcomes are shown in Figure 2. 

Other damage to cardiac tissue includes decreased platelets, phospholipids and endothelial cells, and may activate neutrophils or promote thrombosis with subsequent tissue damage or fibrosis in the heart^25^. Sarcopenia is present^19^. Cardiometabolic alterations promote higher concentrations of saturated monosaccharides, lower concentration of polysaccharides and a lower ratio between saccharides^30^, Increased LDL, ALT, AST and bilirubin, with reduced GFR^26^ and glycemic resistance observed by the TyG index^20^. Cirrhosis, macular degeneration, chronic kidney disease, lupus and arthritis may also be outcomes of the syndrome associated with the potentiation of metabolic disorders^23^.

Studies have shown that COVID-19 patients who were hospitalized or required ICU care were at increased risk of experiencing and being hospitalized for post-COVID-19 cardiac events^25^. Consequently, in severe COVID-19, change in body mass index is independently associated with risk in healthy individuals^18^. Furthermore, the metabolic profiles of community cases with asymptomatic COVID-19 were notably different from those with longer disease, exhibiting an atherogenic lipoprotein phenotype, and this difference was apparent regardless of whether the disease was caused by COVID-19 or another acute event^30^. Finally, there were multiple manifestations of cardiac complications, and many can last for months and even years^27^.

## Discussion

The clinical manifestations of COVID infection affect healthy people and those with cardiometabolic disease (CMD) in the same way; however, the markers and progression of the disease tend to evolve to severe cases when associated with CMD with a greater chance of having "prolonged COVID"^32^. This research identified the elements responsible for the effusion of metabolic worsening after COVID-19 infection and influenced the emergence of new comorbidities.

Obesity is a risk factor for infections associated with severe cases of post-COVID-19 and other respiratory infections^33^. In the study in question, an association was observed between body fat markers and glycemic and lipid indices that evolve to post-COVID conditions. There is evidence that adipose tissue dysfunction and hyperglycemia play a significant role in the clinical course of the disease, but there are still gaps regarding the etiology, epidemiology and treatment of post-COVID syndrome^34^.

As an alternative to tissue damage resulting from cardiometabolic influence, the use of a low-calorie ketogenic diet may be considered in patients with severe obesity for rapid weight loss and during rehabilitation. The high-fat diet may have an anti-inflammatory effect and may be useful for reducing viral replication^33^. 

Furthermore, studies have shown that COVID-19 patients suffer from post-COVID-19 fatigue syndrome, which mainly manifests as chronic fatigue, sleep disturbances, cognitive impairment, muscle pain, and depressive symptoms^35^–^37^. These symptoms are related to an increased resistance to cerebrospinal fluid (CSF) outflow, which leads to congestion of the lymphatic system and promotes the accumulation of toxic substances in the nervous system^36^. 

Elevated levels of cytokine markers may be responsible for pulmonary fibrosis and, consequently, prolonged COVID-19^31^. This cytokine storm affects important organs such as the pancreas. The β cells of pancreatic tissues may be adversely attacked and develop DM due to COVID-19^17^. 

Components of the metabolic syndrome may stimulate dysregulation of the Renin-Angiotensin-Aldosterone System, modulated by ACE-2, leading to increased presence of Angiotensin II. Binding angiotensin II to angiotensin II type I receptors may be a stimulus for cardiovascular insults, such as endothelial dysfunction, thrombosis, and chronic inflammation^21^. The hyperinflammatory state and altered cardiorespiratory function result in excessive fatigue or post-exertional malaise that is being reported in patients with long-COVID. 

The potential use of high-flow oxygen and CPAP are viable alternatives for patients with severe cases of residual symptoms 6 to 12 weeks after discharging due to hypercytokinemia^28^. Hypoxia has also been observed, causing exacerbation of COVID-19 through lipid and glucose disturbances^34^,^38^. These indicators confirm the need to further explore the effects of long COVID. 

In addition to nonspecific symptoms of infection (fatigue, headache, joint pain, myalgia, decreased appetite, and fever), respiratory manifestations – tachypnea, chest pain, and dyspnea – were frequent in outpatients with acute COVID-19. In contrast, fatigue, daytime tiredness, hyposmia/anosmia, taste alteration, and tachypnea were substantially delayed in long COVID-19 participants, and concentration and memory deficits represented predominant manifestations of long COVID-19^21^. 

It is noteworthy that patients with long-term COVID-19 report persistent and debilitating symptoms that affect recovery, quality of life, and broader economic and social activities. Patient experiences are broad, and there is a need for longitudinal approaches to determine the prevalence and fluctuation of symptom exacerbation^39^. However, the data presented here support a clear association between cardiovascular disease and COVID-19. Cardiac injury appears to be common in patients with severe COVID-19, and the long-term cardiovascular damage remains unclear but is known to lead to chronic effects^40^. 

The persistence of the virus in organs such as blood (perivascular inflammation), intestine (changes in the microbiota) and adipose tissue (increased inflammatory response in obese patients and changes in the pathology of diabetes mellitus) increases complications^34^. The study in question observed several implications in the circulatory system. Cardiac inflammation, increased blood pressure, severe chronic fatigue, palpitations, chest pain, shortness of breath and dysautonomia occur^40^. In addition to myocardial injury, myocarditis, arrhythmias, acute coronary syndrome and venous thromboembolism in the months following COVID-19 infection, cardiac symptoms such as atypical chest pain, palpitations and dyspnea and exhaustion are reported^39^. 

Cardiovascular consequences include increased resting heart rate, tachycardia, palpitations, hypotension, syncope, discontinuous flushing, orthostatic tachycardia, newly diagnosed hypertension, angina pectoris, and heart attack. In addition, it increases the risk of developing diabetes mellitus^31^. Long-term COVID can affect all patients with COVID-19 with severity, especially those with cardiometabolic diseases, and may have a worse outcome. There may be several clinical symptoms and manifestations, many of which are nonspecific and have an uncertain epidemiology.

Other presentations with indirect implications on cardiometabolic patterns have also been found. Mental disorders resulting from anxiety disorders, mood disorders, musculoskeletal disorders, neurological conditions (nervous system disorders) and respiratory disorders (asthma) are presented^22^,^23^. There is also muscle dysfunction causing weakness and fatigue leading to loss of muscle mass, a process known as sarcopenia that implies malnutrition and limited physical activity^38^. 

Regarding COVID-19 variants, the prolonged presence of SARS-CoV-2 RNA^30^ studies show that Delta caused high levels of anxiety, insomnia, cognitive impairment, epilepsy or seizures and ischemic strokes, while Omicron caused an increased risk of dementia, mood disorders and nerve and plexus disorders^22^,^23^. In addition, it can cause chronic kidney disease, lupus and arthritis^23^. 

Therefore, it is recommended that healthcare systems be prepared to receive an increasing number of patients with MS-related conditions, given the likely influence of long COVID. People with previous cardiometabolic diseases develop severe weakness and fatigue, and COVID-19 infection is even more likely to cause "long COVID"^40^. 

The findings highlight the continued importance of preventing SARS-CoV-2 infection from progressing to severe disease to reduce potential long-term cardiovascular complications. Thus, it is reinforced that vaccination is the only way to prevent long COVID^31^. 

As limitations, there is a lack of studies involving the implications of cardiometabolic factors after long-term COVID-19 on health. The uncertainties arising from the advances in the clinical picture led to the need for periodic reviews of the topic. The process of searching and selecting studies may have omitted potential studies from the sample that did not contain expressions in the title or abstract of the content that answered the research question. 

## Conclusions

The studies reveal the presence of cardiometabolic markers in post-COVID-19 conditions, which highlights the importance of gathering this scope of knowledge as a tool to verify the implications caused by the infection of the disease in the short, medium and long term. The novelty of the topic reveals weaknesses in conducting research with a specific focus on what was addressed and still generates little evidence on the subject. In summary, considerations are presented regarding the metabolic alteration involving triglycerides, insulin resistance, central obesity resulting in thromboembolism, arrhythmia, heart failure, stroke and coronary disease. 

The summarized data point to the challenge of changing diagnostic and prognostic therapeutic standards related to long Covid, especially its clinical consequences. New studies should advance the knowledge summarized here with developments in the clinical condition of people with long Covid.
